# Multi-Class Motor Imagery EEG Decoding for Brain-Computer Interfaces

**DOI:** 10.3389/fnins.2012.00151

**Published:** 2012-10-09

**Authors:** Deng Wang, Duoqian Miao, Gunnar Blohm

**Affiliations:** ^1^Department of Computer Science and Technology, Tongji UniversityShanghai, China; ^2^Key Laboratory of Embedded System and Service Computing, Ministry of EducationShanghai, China; ^3^Centre for Neuroscience Studies, Queen’s UniversityKingston, ON, Canada; ^4^Canadian Action and Perception NetworkToronto, ON, Canada

**Keywords:** electroencephalogram, brain-computer interface, multi-class motor imagery, artifact processing, EEG channel selection

## Abstract

Recent studies show that scalp electroencephalography (EEG) as a non-invasive interface has great potential for brain-computer interfaces (BCIs). However, one factor that has limited practical applications for EEG-based BCI so far is the difficulty to decode brain signals in a reliable and efficient way. This paper proposes a new robust processing framework for decoding of multi-class motor imagery (MI) that is based on five main processing steps. (i) Raw EEG segmentation without the need of visual artifact inspection. (ii) Considering that EEG recordings are often contaminated not just by electrooculography (EOG) but also other types of artifacts, we propose to first implement an automatic artifact correction method that combines regression analysis with independent component analysis for recovering the original source signals. (iii) The significant difference between frequency components based on event-related (de-) synchronization and sample entropy is then used to find non-contiguous discriminating rhythms. After spectral filtering using the discriminating rhythms, a channel selection algorithm is used to select only relevant channels. (iv) Feature vectors are extracted based on the inter-class diversity and time-varying dynamic characteristics of the signals. (v) Finally, a support vector machine is employed for four-class classification. We tested our proposed algorithm on experimental data that was obtained from dataset 2a of BCI competition IV (2008). The overall four-class kappa values (between 0.41 and 0.80) were comparable to other models but without requiring any artifact-contaminated trial removal. The performance showed that multi-class MI tasks can be reliably discriminated using artifact-contaminated EEG recordings from a few channels. This may be a promising avenue for online robust EEG-based BCI applications.

## Introduction

Electroencephalography (EEG) of brain activity has long been used for clinical diagnosis and exploring brain function. Over the past two decades, EEG-based brain-computer interfaces (BCIs) have received increased attention mainly due to the ease of use, high temporal resolution, and low cost compared to other non-invasive measurements of brain activity, such as fMRI, MEG, PET scans, etc (Wolpaw et al., 2002; Noirhomme et al., 2008; Arvaneh et al., 2011). Motor imagery (MI), which can be defined as the mental rehearsal of a motor act without overt movement execution (Alkadhi et al., 2005), is often used in EEG-based BCIs. By thinking about moving their arms, hands, tongue, legs, or rotating an object, participants can produce relevant motor-related EEG patterns. If properly decoded, these patterns can then be translated into a command to control external devices like a mobile robot/wheelchair (Millán et al., 2004; Lew et al., 2006), or a virtual helicopter (Doud et al., 2011).

However, there are serious challenges. For example, low signal decoding performance, highly subject-specific data, and low processing speed limit the practical applications as well as usefulness in analyzing neurophysiological data for human brain investigations. One reason for this is that EEG signals are prone to contamination from artifacts such as blinking or movements of the eyes (electrooculography, EOG), heart beats (electrocardiography, ECG/EKG), and electromyography (EMG) activity of cranial musculature. Movements of head, body, jaw, or tongue, etc. can also interfere with recordings. For example, EOG artifacts are a major noise source in EEG recordings. However, restricting eye movements/blinks limits experimental designs and may impact cognitive processes under investigation (Joyce et al., 2004). Generally speaking, there are two kinds of strategies for obtaining high-quality EEG recordings (Joyce et al., 2004; Fatourechi et al., 2007; Schlögl et al., 2007; Hallez et al., 2009; Zhou and Gotman, 2009): (1) eliminating contaminated trials after visual inspection, or (2) correcting the artifacts automatically. The former method leads to a substantial loss of data. Moreover, it requires EEG experts to carefully inspect each trial, a process that is generally very time-consuming and subjective. On the other hand, regression-based techniques have shown promising results in the field of EOG-related artifact correction (Schlögl et al., 2007). Another effective automatic method to correct for EOG artifacts is independent component analysis (ICA; Joyce et al., 2004; Fatourechi et al., 2007; Hallez et al., 2009; Zhou and Gotman, 2009). In this study, we combine regression analysis (RA) with ICA to automatically recover the source signals from EEG signals contaminated by EOG as well as artifacts generated by other sources.

Another important challenge for online EEG decoding is choosing the optimal number of electrodes and the relevant frequency bands to improve discrimination between MI (or other) tasks. In principle, using a small number of channels without carefully choosing their locations may cause a loss of important electrophysiological information. However, including more channels to collect data will provide redundant information which could increase the risk of data over-fitting, and which increases computational complexity to the degree that would make real-time BCI application infeasible with currently available desktop computer power. Therefore, it is necessary to determine the optimal channel set in EEG-based BCI studies. Moreover, the optimized electrode locations, obtained through what is also known as spatial pattern filtering, would reflect the specific motor cortical regions related to different MI tasks which helps to provide further insight into cognitive resources used in the tasks. Pfurtscheller and Aranibar (1977, 1979); Pfurtscheller and Lopes da Silva (1999) introduced an event-related (de-) synchronization (ERD/ERS) analysis method to distinguish between channels and to select a channel set for MI classification. However, the ERD/ERS depends on frequency band and so selecting the most discriminating frequency bands is important for ERD/ERS analysis. In Pfurtscheller and Lopes da Silva (1999), the authors suggested three effective ways to determine the upper and the lower limits of the band-pass filter, i.e., detect the frequency bands based on short-time power spectra, continuous wavelet transform, and peak frequency. In this study, we assume that non-contiguous frequency band filters might provide a much more accurate way to quantify the sensorimotor in the frequency domain than manually selecting a broad frequency range filter. Hence, we conducted an automatic selection of subject-specific reactive non-contiguous frequency bands via state-of-the-art information theoretic sample entropy.

To summarize, the principal aim of this study was to introduce a novel multi-class MI EEG decoding for BCIs, including an automatic artifact correction method to recover the original source signals from EOG and other artifacts and choosing the least number of channels yet yielding the best performance with the most reactive frequency bands, i.e., sub-bands in frequency domain sets, of the recordings. We tested the performance of our method on a well-known publicly available data set from BCI competition IV in 2008.

## Data Acquisition and Datasets

In this study, we used dataset 2a from BCI Competition IV^1^, i.e., a four-class MI study which was provided by the Institute for Knowledge Discovery (Laboratory of Brain-Computer Interfaces), Graz University of Technology (Austria). Compared to datasets from past BCI Competitions (BCI Competition I, announced at NIPS 2001, BCI Competition II, also called BCI Competition 2003, and BCI Competition III, i.e., BCI Competition 2005), there were eye movement artifacts in dataset 2a as a new challenging problem that is highly relevant for practical BCI systems.

**Figure 1:**
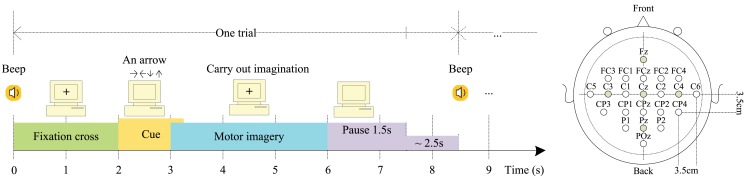
**Left: trial timing of the motor imagery paradigm**. (Cued MI: left hand, right hand, both feet, tongue); Right: electrode montage corresponding to the international 10–20 system (adapted from Brunner et al., 2008).

The data set consists of EEG data from nine subjects. Each subject was sitting in a comfortable armchair in front of a computer screen. The cue-based BCI paradigm consisted of four different MI tasks, namely the imagination of movement of the left hand (class 1), right hand (class 2), both feet (class 3), and tongue (class 4). Two sessions on different days were recorded for each subject. Each session was comprised of six runs separated by short breaks. Each run consisted of 48 trials (12 for each of the four possible classes), yielding a total of 288 trials per session. On the left of Figure 1 is depicted the timing scheme of one trial. An acoustic stimulus indicated the beginning of a trial and a fixation cross (+) was displayed for 2 s, which subjects were requested to fixate. Then a cue in the form of an arrow pointing either to the left, right, up, or down (corresponding to one of the four classes mentioned above) was displayed for 1.25 s. This prompted the subjects to carry out the mental imagination until the fixation cross disappeared from the screen at *t* = 6 s. A short break followed which lasted 1.5–2.5 s allowing subjects to relax. Twenty-two referenced EEG channels (Figure 1 right) and three monopolar EOG channels (positioned above the nasion and below the outer canthi of the eyes) were recorded using Ag/AgCI electrodes (left mastoid serving as reference and the right mastoid as ground), were sampled at 250 Hz and band-pass filtered between 0.5 and 100 Hz, with the 50 Hz notch filter enabled (Brunner et al., 2008).

## Analysis Methods

**Figure 2:**
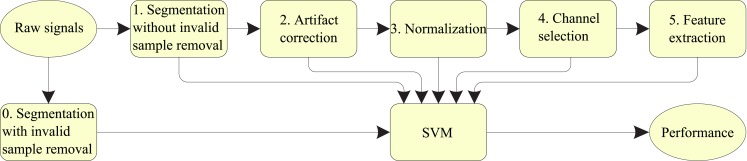
**Schematic diagram for the EEG signal processing procedure (Five-stage decoding EEG – FSDE)**.

We labeled the proposed method a five-stage decoding of EEG (FSDE). First, the original EEG signals were segmented into trials according to the header structure information (see Brunner et al., 2008 for details). Then, the correction method based on RA in combination with the fast ICA (FastICA) was used. The third stage was a normalization process. The *z*-score normalization was applied on the EEG segments. The fourth stage consisted of channel selection. After comparing the ERD/ERS value between target MI class and other non-target classes, data from the selected channels were used for later feature extraction. Finally, the classification was performed using support vector machine (SVM) classifiers. The full processing procedure is shown schematically in Figure 2, and the details are explained in the following sub-sections.

### Segmentation

As Figure 2 shows, there are two different strategies in EEG segmentation. One is segmentation without invalid sample removal. The other is segmentation with artifact removal. Since our goal was to develop an algorithm that was robust to outliers and artifacts, no trials were removed in our experiment.

### Artifact correction

Extending the work of Schlögl et al. (2007), we assume the following linear mode including EOG and other artifacts:

(1) X=AS+UK,

where $X=\left[x_{1},x_{2},\cdots \;,x_{N}\right]\in {\mathbb{R}}^{N\times T}$ denotes a matrix that represents the recorded EEG signals, *N* and *T* denote the number of channels and the number of sampled time-points, respectively, *A* is composed of constant coefficients *a_ij_* and is a linear mixture unknown matrix, *S* is the uncontaminated signal without artifact contamination, *U* denotes the three EOG components, and *K *= [*k*_1_(*i*), *k*_2_(*i*), *k*_3_(*i*)]^T^ indicates the weights of the EOG artifacts at EEG channel *i* for signal correction.

Let *Y* = *AS*, then Eq. 1 can be written as

(2) Y = X-UK.

In order to obtain *Y*, the EOG noise *U* and its weighting coefficients *K* must be known. Here, *U* is known because it was recorded by separate EOG channels (they are positioned close to the eyes in order to minimize the influence of non-EOG components). In order to identify the weighting coefficients *K*, we assume that the EEG signal and the EOG noise are independent because they come from different cognitive component sources, then

(3) $$<U^{T}Y>=<U^{T}X>-<U^{T}U>K$$

where $<U^{T}Y>\;=\;0$ results in

(4) $$K=<U^{T}U{>}^{-1}<U^{T}X>$$

where $<U^{T}U>$ is the auto-covariance matrix of the EOG channels, and $<U^{T}X>$ is the cross-covariance between the EEG and EOG channels. Accordingly, the output *Y* can be calculated from EOG artifacts by Eq. 2.

Our aim is to obtain the independent source signals *S* which cannot be recorded directly. Therefore, after correcting EEG from EOG artifacts, ICA was employed to unmix the signals from other artifacts. Those components corresponding other artifacts are not identified via visual inspection but will be discarded through the subsequent channel selection algorithm. In this study, 25 physical sources emit electric signals. Each records a mixture of the original source signals.

(5) $$Y=AS\Rightarrow \left[\begin{array}{c} y_{1}\left(t\right)\\ \vdots \\ y_{n}\left(t\right) \end{array}\right]=\left[\begin{array}{ccc} a_{11} & \cdots & a_{1n}\\ \vdots & \ldots & \vdots \\ a_{n1} & \cdots & a_{nn} \end{array}\right]\;\left[\begin{array}{c} s_{1}\left(t\right)\\ \vdots \\ s_{n}\left(t\right) \end{array}\right],$$

where $y_{i}\in Y,\;a_{ij}\in A,\;s_{i}\in S$.

All signals can be regarded as a linear superposition of the real task-related brain signals *S*. The aim is to find the source signal *S* from the mixture *Y*. Since the mixing coefficients *a_ij_* are different enough to make the matrix invertible, there exists a matrix *W* with coefficients *w_ij_*. Multiplying the unmixing matrix *W* to Eq. 5, results in

(6) $$Z=WY=WAS\Rightarrow \left[\begin{array}{c} z_{1}\left(t\right)\\ \vdots \\ z_{n}\left(t\right) \end{array}\right]=\left[\begin{array}{ccc} w_{11} & \cdots & w_{1n}\\ \vdots & \ldots & \vdots \\ w_{n1} & \cdots & w_{nn} \end{array}\right]\left[\begin{array}{c} y_{1}\left(t\right)\\ \vdots \\ y_{n}\left(t\right) \end{array}\right],$$

where *Z *= [*z*_1_, *z*_2_, …, *z*_n_]^T^ can be regarded as being mathematically similar to signal *S*, especially when *WA *= *E*, i.e., matrix *W* and *A* are inverse to one another and *Z* is equal to the source signal *S. W *= [*w*_ij_]is a so-called unknown unmixing matrix.

Based on this, the aim has been changed to estimate *w_ij_* in Eq. 6. In fact, if the signals are not Gaussian, it is sufficient to find an “unmixing matrix” by considering the statistical independence of different linear combinations of *Y*. In the present study, the classical ICA algorithm (FastICA MATLAB pack^2^) was used to determine *W* from the given multidimensional signals (see FastICA in Appendix for detail). Finally, the independent source signal *Z* can be calculated by Eq. 6.

### Normalization

Before normalization, the signals from each electrode were winsorized to reduce the effects of large amplitude outliers (Hoffmann et al., 2008): for the signals from each electrode the 5th percentile and the 95th percentile were computed. Amplitude values lying below the 5th percentile or above the 95th percentile were then replaced by the 5th percentile of the 95th percentile, respectively. We used this method because both mean and SD are sensitive to outliers. Normalization steps were then applied to EEG signals for possible variations in signal acquisition from trial to trial. In our experiments, normalization techniques, such as log (Nakayama and Inagaki, 2006), min-max normalization, and zero-mean normalization (*z*-score) were tested. Compared with other normalization methods, the *z*-score normalization data set had the highest accuracy. The normalized signals S^’^ are given by

(7) $$S_{ijk}^{\prime }=\frac{S_{ijk}-\mu_{ij}}{\sigma_{ij}},$$

where $\mu_{ij}=\frac{1}{P}\overset{P}{\underset{k=1}{\sum }}S_{ijk},\;\sigma_{ij}={\left(\frac{1}{P}\overset{P}{\underset{k=1}{\sum }}{\left(S_{ijk}-\mu_{ij}\right)}^{2}\right)}^{\frac{1}{2}},\;S\in {\mathbb{R}}^{N\times T\times P},$ *N*, *T*, and *P* denotes the number of channels, number of measurement samples, and number of trials, respectively.

### Channel selection based on ERD/ERS analysis

It is well-known that brain rhythms as measured by EEG are time series that composed of mixtures of multiple frequency components, such as δ (1–4 Hz), θ (4–8 Hz), α (8–13 Hz), β (13–30 Hz), and γ (>30 Hz) rhythms. People have naturally occurring brain rhythms over areas of the brain concerned with different functional states. For example, when people imagine moving, the functional connectivity of cortex is changed, i.e., the amplitudes of μ and central β rhythms are first suppressed, then enhanced (Pfurtscheller and Lopes da Silva, 1999). These two changes are called ERD/ERS (event-related desynchronization and event-related synchronization), respectively (Pfurtscheller, 1977, 1992; Pfurtscheller and Aranibar, 1977; Pfurtscheller and Lopes da Silva, 1999). Because different EEG rhythms can distinguish patterns of neuronal activity associated with specific behavioral and cognitive processing functions, different patterns of synchronization or desynchronization could result from different forms of processing or computation in the brain and represent different rhythmic states. The ERD/ERS is defined as the percentage of power decrease (ERD) or power increase (ERS) within a given frequency band relative to a reference interval (Pfurtscheller and Lopes da Silva, 1999). Mathematically, it can be estimated as follows:

(8) $${\text{ERDS}}_{i}^{\left(k\right)}=\frac{A_{i}^{\left(k\right)}-R_{i}^{\left(k\right)}}{R_{i}^{\left(k\right)}}\times 100\%,$$

where $A_{i}^{\left(k\right)}$ represents the power during an experimental task segment of class *k*, channel *i*, and $R_{i}^{\left(k\right)}$ denotes the power of given frequency bands during the reference time segment of class *k*, channel *i*. The value ${\text{ERDS}}_{i}^{\left(k\right)}$ indicates the relative power during the task. A negative value of ${\text{ERDS}}_{i}^{\left(k\right)}$ indicates a power decrease during the stimulation in the frequency band of interest which is a desynchronization. A value of zero means no power change in the interested frequency band, i.e., there is no ERD/ERS phenomenon. Finally, a positive value signifies an increase of power, i.e., synchronization. Furthermore, the larger the ERD/ERS, the more apparent the ERS phenomenon is.

Different rhythms evoked in specific MI tasks involve different brain areas with different mental processes which may produce different brain patterns useful in a BCI. After calculating ERDS for all channels and classes, in order to investigate the EEG pattern changes of motor imageries for each frequency, we proposed an approach based on sample entropy – a modification of the approximation entropy introduced by Richman and Moorman (2000). Sample entropy is employed to measure the uncertainty of the next observation knowing *m* past observations and using a certain resolution *r*. In this approach, the non-contiguous bands consisting of sub-upper and sub-lower limits of the band-pass filter (e.g., if {4–6, 8–12} is a non-contiguous frequency band set, 4 and 8 are called sub-lower limits, 6 and 12 are sub-upper limits) is determined which is much more accurate than a single frequency band for quantifying the sensorimotor rhythms in the frequency domain. See Sample entropy in Appendix for the computation of Sample Entropy.

We wished to calculate the relevant electrode positions (spatial domain) for detection and classification of MI-related EEG patterns in the cortex, and therefore used the following algorithm on the training set to select the optimal channel set with the most reactive multiple frequency bands of the recordings.

As for the test set (also called evaluation data), the normalized EEG signals were filtered using the saved non-contiguous frequency components (including P_i,*_ and γ_i,*_) for each channel *i* on the selected optimal channel set. It should be noted, for each channel the multiple frequency band set is made up of several non-contiguous sub-bands since some frequency components (stop frequency bands) were removed using notch filters. The size of the frequency bands set varies according to electrode placement, and it also varies from subject to subject. For each subject, multiple frequency bands were selected during training stage. Then the optimal channel set with the most reactive multiple frequency bands was applied to test recordings for the same subject.

**Input:** Normalized EEG segments (*S*) with all channels, and the corresponding header structure (*H*).

**Output:** Optimal channel set (*O*).

S1 Set the reference interval from 0 to 2 s (visual cue-onset at second 2).
S2 Calculate ${\text{ERDS}}_{i}^{k}$ for all channels (*i*) and classes (*k*) using Eq. 8 to estimate the power changes caused by MI in specific frequency components from 2 to 40 Hz using the BioSig toolbox^3^ [frequency borders = [2, 40] with 2 Hz bandwidth and in 1 Hz frequency step size, i.e., calculate for the segments: (1–3 Hz), (2–4 Hz), …, (39–41 Hz)]. The classical bandpower method of quantification of ERD/ERS (Pfurtscheller and Aranibar, 1977) was used.
S3 For each channel *i*
  - For each column of ${\text{ERDS}}_{i}^{k}$ (frequency component):
    i Calculate SampEn using a sliding time window of width 2 s from 2.5 to 3.5 s for each class, respectively. This was done since ERD and ERS display some intra- and inter subject variability and are not restricted to 2 s time windows.
    ii Calculate the significant frequency components with the 95% confidence interval by using the paired-sample *t*-test between each combination of two different SampEn which maximizes differences between two classes.
    iii Construct a set of frequency components (*fC*_i_) in which the frequency of each component has a significant difference for all combinations.
  - For each component *p_i,j_* ∈ *fC_i_*:
    i Set the upper limit to *p_i,j_*, and determine the stop frequency band set $\left(\Gamma_{i,j}=\left\{\tau |1<\tau <p_{i,j},\tau \notin fC_{i}\right\}\right)$. *S_i_* passes through a five-order Butterworth low-pass (*p_i,j_ *+ 1) filter and a notch filter for each stop frequency τ.
    ii Calculate classification accuracy using the filtered signal (*fS_i,j_*), save fS_i,*_ which has the best accuracy (*Acc_i_*) for step S6(b), and save the frequency component (p_i,*_) with the corresponding stop frequency band set (Γ_i,*_) which will be used in the *Acc_i_* test session.
S4 Sort all EEG channels into a list in descending order according to *Acc_i_*.
S5 Initialize the size of *O:l *= 0, and the current accuracy: *Acc*(1) = 0.
S6 For *l *= 1:number of EEG channels
  - *l←l+1*.
  - Calculate classification accuracy *Acc*(*1*) using *fS*_1,*_:*fS*_1,*_.
S7 Return the optimal channels set *O*(*1:l*) which presents the highest classification accuracy obtained by validation test on the training set.

### Feature extraction

We used feature extraction to find a suitable representation of the EEG recordings that can be simplified in the subsequent classification. There are a variety of feature extraction methods used in BCI systems (see e.g., Bashashati et al., 2007 for a review). Considering the non-stationary characteristics (rapidly varying over time and particularly across tasks) of MI EEG, two kinds of features were extracted in this study. One was based on the fact that a source active for one mental task is active with a different energy for another mental task (inter-class diversity). The other was based on the fact that motor tasks involve a succession of activations in different brain areas. This method has been applied successfully in Gouy-Pailler et al. (2008). In the current study, the data from the selected channel in the selected frequency bands were separated into 4 timeframes (0.5 s long) from *t* = 2.5 s to *t* = 4.5 s for feature extraction. Afterward, in order to reduce the dimension of the extracted feature, we used principle component analysis (PCA; i.e., eigenvectors with eigenvalues greater than one were chosen). We used a training set $S^{\text{train}}\in {\mathbb{R}}^{T\times N^{\prime }}$, where *T* and *N′* denote the number of sampled time-points and the number of selected channels, respectively. We defined the eigenvectors of the covariance matrix of the training set as follows:

(9) $$\begin{array}{l} V_{i}=\{\text{PCA}\left(S_{\;i}^{\text{train}}\left(t\in \left[2.5,\;4.5\right]\right)\right),\\ \text{PCA}\left(S_{\;i}^{\text{train}}\left(t\in \left[2.5:0.5:\;4.5\right]\right)\right)|i\in \left[1,\cdots ,N^{\prime }\right]\}. \end{array}$$

For the test set $S^{\text{test}}\in {\mathbb{R}}^{T\times N^{\prime }},$ the feature projection matrix is determined by,

(10) $$F^{\text{test}}=S^{\text{test}}\cdot V.$$

### Classification

After the feature extraction, the feature vectors are subjected to a classifier. SVMs (Vapnik, 1995) introduced in 1995 are some of the most frequently used machine learning methods both for classification and regression, and have proven to be useful in EEG signal classification for MI and BCI applications (Ang et al., 2008; Noirhomme et al., 2008; Arvaneh et al., 2011). To verify our method, SVMs with radial basis kernel function as the classifier was employed by implementing the LIBSVM toolbox^4^ (Chang and Lin, 2001). Because of individual differences, training sets (session 1: A01T to A09T) from the nine subjects (Subject 1 to Subject 9) were used to training the SVM classifier. To limit the amount of over-fitting and reduce training time, we used a 10-fold cross-validation procedure, where 90% of all trials in each file were used for the training set, and the remaining trials were used for validation to determine performance. This was repeated 10 times for different partitions of the training set. After the classifiers had been trained from the training sets, they were applied to the data sets (session 2: A01E to A09E). Therefore, the channels and frequency bands were selected based on the cross-validation accuracy from the session 1.

## The Performance Evaluation Methods

Classification accuracy, kappa score, and information transfer rate (ITR) in bits/trial were calculated for performance evaluation of the proposed method.

### Classification accuracy

Classification accuracy was measured according to (11) to evaluate the performance of the proposed methods. This criterion was also used for selecting electrode locations, and frequency bands in section III.

(11) $$\text{Accuracy}=\left(\frac{N_{\text{correct}}}{N_{\text{total}}}\right)\times 100\%,$$

where *N*_correct_ is the number of correct classified samples, and *N*_total_ is the number of total samples to be classified (the test set).

### Kappa score

In order to compare our results with previous results reported by BCI Competition IV^5^, the kappa score as well as the classification accuracy were calculated. Cohen’s kappa score often simply called Kappa score is thought to be a robust statistical measure for qualitative categorical items. The value of the Kappa ranges between 1 and −1, where 1 corresponds to perfectly correct classification and −1 to completely erroneous classification, while a Kappa score of 0 corresponds to chance performance. The equation defined in Cohen (1960) is

(12) $$k=\frac{\mathrm{Pr}\left(a\right)-\mathrm{Pr}\left(e\right)}{1-\mathrm{Pr}\left(e\right)},$$

where Pr(*a*) is the relative observed agreement among raters, and Pr(*e*) is the hypothetical probability of chance agreement, using the observed data to calculate the probabilities of each observer randomly saying each category.

### Information transfer rate

Since speed for assessing this kind of non-invasive communication and control systems (BCIs) would be affected by the characteristics of the specific application which make the comparisons between different studies difficult, a common method which incorporates speed and accuracy in a single value is ITR, or bit rate (Wolpaw et al., 2002). This measure is calculated by,

(13) $$B=\log_{2}N+P\log_{2}P+\left(1-P\right)\log_{2}\left(\frac{1-P}{N-1}\right),$$

where *P* is the classification accuracy, i.e., how well thoughts are recognized, and *N* is the number of mental tasks.

## Results and Discussion

**Figure 3:**
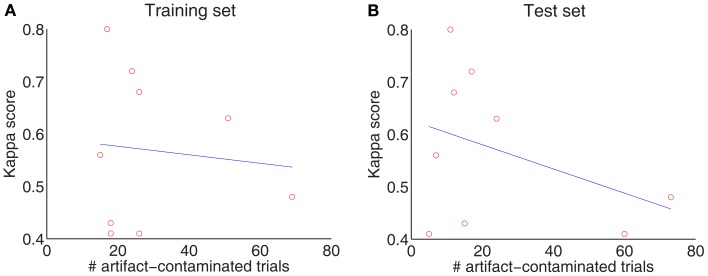
**Influence of artifacts: the number of artifact-contaminated trials influences the decoding of the test set (A) but not the training set (B)**.

Although a visual inspection of the raw EEG data was performed by an expert (see Table 1), no trials with marked artifacts were removed in this study so that we could evaluate the system’s robustness and sensitivity to outliers and artifacts, as in Gouy-Pailler et al. (2010). Figure 3 plots the correlation between the number of artifact-contaminated trials and kappa score for the training and test sets, showing that kappa scores are influenced more by artifact-contaminated trials from the test sets than from the training sets.

**Table 1:** **Summary of the number of artifact-contaminated trials for each subject**.

Subjects	Files	Total	Artifact-contaminated
S1	A01T	288	15
	A01E	288	7
S2	A02T	288	18
	A02E	288	5
S3	A03T	288	18
	A03E	288	15
S4	A04T	288	26
	A04E	288	60
S5	A05T	288	26
	A05E	288	12
S6	A06T	288	69
	A06E	288	73
S7	A07T	288	17
	A07E	288	11
S8	A08T	288	24
	A08E	288	17
S9	A09T	288	51
	A09E	288	24

Each trial lasted for 6 s, but not all time-points of this 6 s period carry information about the difference among the four MI tasks. Subjects were told to begin imagining after the execution cue was presented but they could have begun imagining right after the presentation of the preparation cue. Therefore, the EEG data from 0.5 to 2.5 s after the visual cue (i.e., from 2.5 to 4.5 s, see Figure 1) were used in this study. We used the exact same time window for the channel selection algorithm, feature extraction with PCA, and classification. The selected time segment was also used by the winner of the BCI competition IV dataset 2a (Ang et al., 2008). Since the frequency bands of interest vary from subject to subject (Pfurtscheller et al., 1997, 2000), we used a subject-specific strategy in this study.

**Figure 4:**
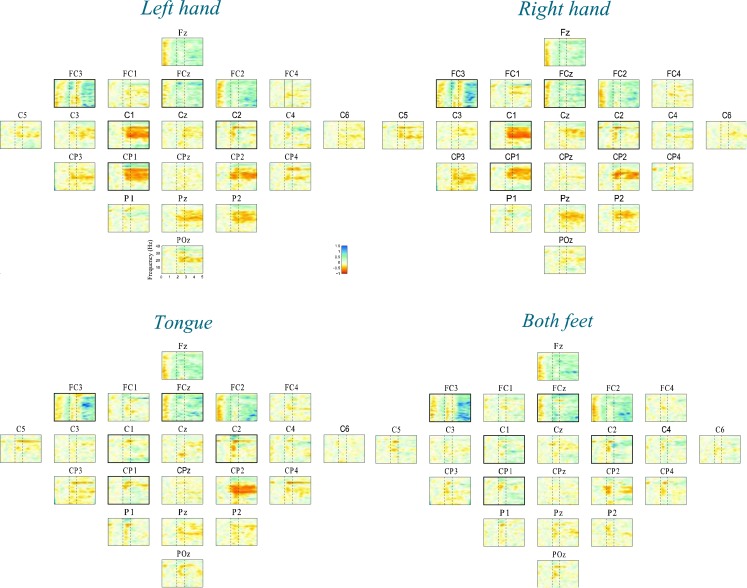
**Time-frequency maps (ERD/ERS) relative to the baseline recorded seconds before the event for all 22 EEG channels during four-class motor imagery task**. Event-related desynchronization (ERD) is plotted in red, while event-related synchronization (ERS) is in blue.

**Figure 5:**
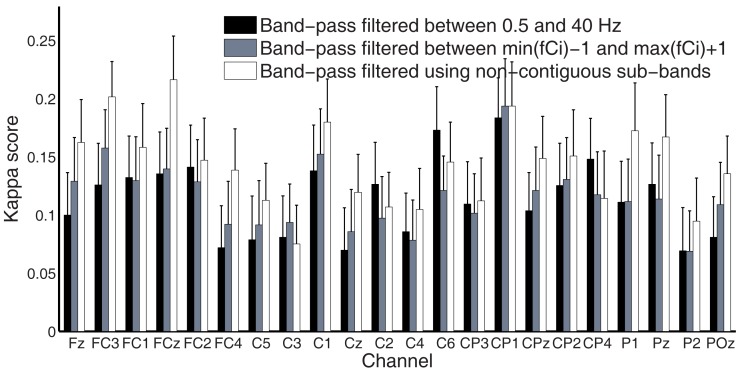
**Comparison of kappa scores using three different filtering approaches**.

Time-frequency maps can provide an overview of the activity over broad frequency ranges and electrode locations showing significant band power increases or decreases during MI tasks. Figure 4 shows an example of ERD/ERS map calculated from 22 EEG channels during imagery of movements of the left, right hand, tongue, or both feet. The maps cover the frequency range from 2 to 40 Hz, which is sufficient to detect important ERD/ERS patterns such as μ and β rhythms. The reference period was 0 to 2 s. It is not easy to detect differences through visual inspection even though the selected channels were highlighted by bold boxes. In order to quantify significant ERD/ERS changes, we used paired-sample *t*-tests to calculate the significant difference based on sample entropy among all frequency bands, thus selecting the optimal channels for discrimination among four MI tasks. Figure 5 compares the performance between the 0.5 and 40 Hz band, the [min(*fC*_i_)−1]−[max(*fC*_i_)+1] band, and the proposed non-contiguous frequency sub-band filter approach for each EEG channel across all nine subjects which demonstrates effectiveness of the non-contiguous approach proposed for most channels.

### Optimum channel selection

EEG signals are electrical activity recorded from multiple electrodes placed on the surface of scalp and generally, not all signals from all electrodes are related to the desired task. This means that each channel makes a particular contribution to the discrimination between BCI tasks. Some are highly discriminative, some low. In addition, when we consider computational complexity and time costs, some channels should be discarded. In our channel selection algorithm, two input parameters must be specified to compute sample entropy. One is the embedding dimensions *m*, the other is the resolution tolerance *r*. Although both are critical in determining the outcome of this method for entropy estimation, no guidelines exist for optimizing their values. The various existing rules generally lead to the use of values of *r* between 0.1 and 0.25 and values of *m* of 1 or 2 for data records of length N ranging from 100 to 5,000 data points (Pincus, 1991; Lake et al., 2002). In our experiment, parameter values *m *= 2 and *r *= 0.2 times the standard deviation of the original data sequence were chosen.

**Figure 6:**
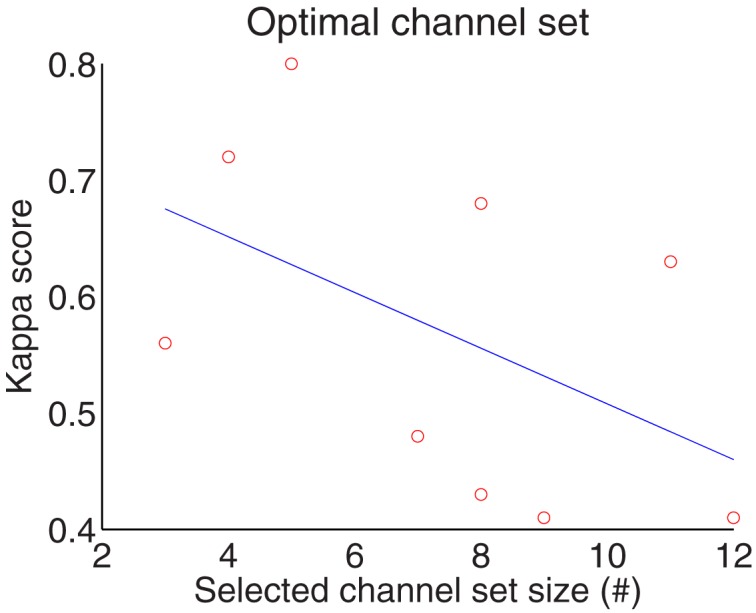
**Correlation between the selected channel set size and kappa score**.

According to Table 2, the optimum number of selected channels is in the range of 3–12, with an overall mean of eight which significantly reduces the number of channels from 22. The number of channels for subject 5 and 8 are 3, the smallest number amongst the subjects, while the largest number of selected channels is 12 for subject 6. Figure 6 gives correlation between the selected channel set size and kappa score. It shows kappa score is influenced by the size of the channel set. Moreover, the results in Table 3 shows that the third stage (channel selection) yields superior averaged test accuracies of 71.83 ± 6.76% and 64.76 ± 6.85% for session 1 and session 2 with the use of only 3–12 from 22 channels.

**Table 2:** **Summary of selected channels with the highest classification accuracy (Acc) for each subject**.

Subjects	Number (selected channels)	Acc (%)
S1	8{CP2 C2 P2 FC1 FC2 CP3 P1 POz}	71.43
S2	11{C2 FC3 C1 P1 C4 C5 FC2 Cz CP2 P2 CPz}	67.50
S3	6{CP3 CP2 CPz P2 C3 CP4}	64.29
S4	9{CP2 CP1 Cz FC2 Pz C3 FC1 CP4 FCz}	57.50
S5	3{FC2 P1 C6}	87.86
S6	12{C1 C5 FC3 C6 CP2 POz Pz FCz CP4 C3 Fz FC2}	58.93
S7	11{FC3 FCz FC2 C2 Cz C6 FC1 CP4 CPz POz P2}	85.71
S8	3{FC4 CP1 FCz}	79.29
S9	11{C4 C6 CPz C2 FC4 C5 CP1 P2 Fz Pz Cz}	73.93

**Table 3:** **Comparison of 10-fold cross-validation classification accuracy (%) for each processing stage presented in this paper for each subject on the training set (session 1) and test set (session 2)**.

Subject	Segmentation		Artifact correction		Normalization		Channel selection		Feature extraction	
	Mean	Std.	Mean	Std.	Mean	Std.	Mean	Std.	Mean	Std.
S1/A01T	22.86	6.78	52.86	6.25	57.14	9.96	71.43	8.25	71.79	9.29
S2/A02T	27.14	3.84	57.5	11.1	61.07	10.97	67.5	7.98	66.79	7.15
S3/A03T	23.93	5.34	41.43	4.82	43.93	7.91	64.29	4.45	65.36	5.06
S4/A04T	24.64	2.64	40.71	9.55	41.43	9.4	57.5	7.61	61.43	7.3
S5/A05T	26.07	3.78	61.07	7.61	70.71	6.48	87.86	6.78	89.29	7.53
S6/A06T	22.14	6.48	44.29	7.38	53.57	9.82	58.93	4.84	62.14	6.98
S7/A07T	25.71	2.26	76.07	8.08	81.07	9.97	85.71	6.73	84.64	5.6
S8/A08T	28.93	7.42	49.29	8.04	72.14	4.05	79.29	4.99	79.29	6.25
S9/A09T	23.21	5.39	54.29	8.72	64.29	9.96	73.93	9.23	79.29	8.38
**Mean**	24.96	4.88	53.06	7.95	60.6	8.72	71.83	6.76	73.33	7.06
S1/A01E	18.21	2.64	47.14	7.68	49.29	10.88	56.07	5.34	59.29	7.18
S2/A02E	25.71	5.27	43.57	10.62	49.29	7.1	55	7.75	59.29	10.41
S3/A03E	20.71	7.1	52.86	9.04	53.93	9.44	52.86	7.86	57.5	8.49
S4/A04E	17.5	4.28	32.14	10.91	42.14	14.95	45.71	9.34	55.36	6.99
S5/A05E	26.07	2.94	31.07	8.92	36.79	7.35	80	6.56	76.07	8.08
S6/A06E	15.71	3.84	28.93	7.98	49.29	12	56.79	8.82	56.07	10.11
S7/A07E	27.86	2.82	77.86	7.3	85.71	5.58	85.71	5.58	83.93	6.57
S8/A08E	31.43	5.27	55.36	10.55	71.79	9.59	74.29	4.05	76.07	5.34
S9/A09E	13.93	4.89	60.71	8.91	67.86	9.52	76.43	6.34	75.71	7.3
**Mean**	21.9	4.34	47.74	9.1	56.23	9.6	64.76	6.85	66.59	7.83

### 10-fold cross-validation for each session data

In order to evaluate each stage’s performance of the processing framework, we used a 10-fold cross-validation on the two sessions (i.e., the training set and the test set which were recorded on two different days), respectively. The detailed view of classification accuracies of all subjects for each stage is summarized in Table 3. As seen from this table, average accuracy improves at each stage across subjects for each session. For session 1 and session 2, the proposed artifact correction algorithm yielded an average improvement of 28.1 and 15.8% in classification accuracy. Similarly, the proposed channel selection algorithm yielded an average improvement of 11.2 and 8.5% in the classification accuracy. From Table 3, we can see that the proposed artifact correction algorithm and the channel/frequency band selection algorithm yielded better performance which show these two stages made the biggest contribution to our method. The parameters of the algorithm were estimated only on the session 1 and were used on the following session-to-session transfer test.

### Comparison to previous work

To compare with the results of the winners of dataset 2a of BCI Competition IV, we used session-to-session transfer using the same criterion, namely, the kappa score. The procedure of this evaluation method is greatly simplified, using the first session (A01T to A09T) as training data (also called calibration data) to find the optimal parameters and then to apply the procedure to unseen data (also called evaluation data; session 2: A01E to A09E) to test performance. This is the most meaningful performance measure in actual BCI experiments. Table 4 summarizes the comparison of the kappa score of the proposed method with the existing multi-class methods for each subject on the dataset 2a of BCI competition IV. It can be seen that our proposed method (FSDE) without artifact removal performed comparably to the best competitors^5^ and Gouy-Pailler et al. (2010). Our experimental results also found that frequency 1 Hz is important only for subject 5 and 6. That is to say, if the signals from subject 5 and 6 were filtered above 1 Hz, the kappa scores were very low. We believe that this is why the three best competitions 1st–3st had the lowest results for these two subjects (see Table 4). For the competition winner, signals were band-pass filtered into multiple frequency bands (4–8, 8–12, …, 36–40 Hz), for the competition second ranked, signals were band-pass filtered between 8 and 30 Hz, and the authors of the third ranked paper filtered signals in an 8–25 Hz band.

**Table 4:** **Comparison of session-to-session transfer performance for each subject**.

Subject	Training set	Test set	1st	2st	3st	MSJAD	FSDE
S1	A01T	A01E	0.68	**0.69**	0.38	0.66	0.56
S2	A02T	A02E	**0.42**	0.34	0.18	**0.42**	0.41
S3	A03T	A03E	0.75	0.71	0.48	**0.77**	0.43
S4	A04T	A04E	0.48	0.44	0.33	**0.51**	0.41
S5	A05T	A05E	0.4	0.16	0.07	0.5	**0.68**
S6	A06T	A06E	0.27	0.21	0.14	0.21	**0.48**
S7	A07T	A07E	0.77	0.66	0.29	0.3	**0.8**
S8	A08T	A08E	**0.75**	0.73	0.49	0.69	0.72
S9	A09T	A09E	0.61	**0.69**	0.44	0.46	0.63
**Mean**			0.57	0.51	0.31	0.5	0.57
**Std**.			0.18	0.23	0.15	0.18	0.15

*Kappa Scores obtained by three best competitions 1st–3st (see text footnote 5), MSJAD (Gouy-Pailler et al., 2010), as well as the method (FSDE) presented in this paper. Best kappa values are highlighted in bold*.

**Figure 7:**
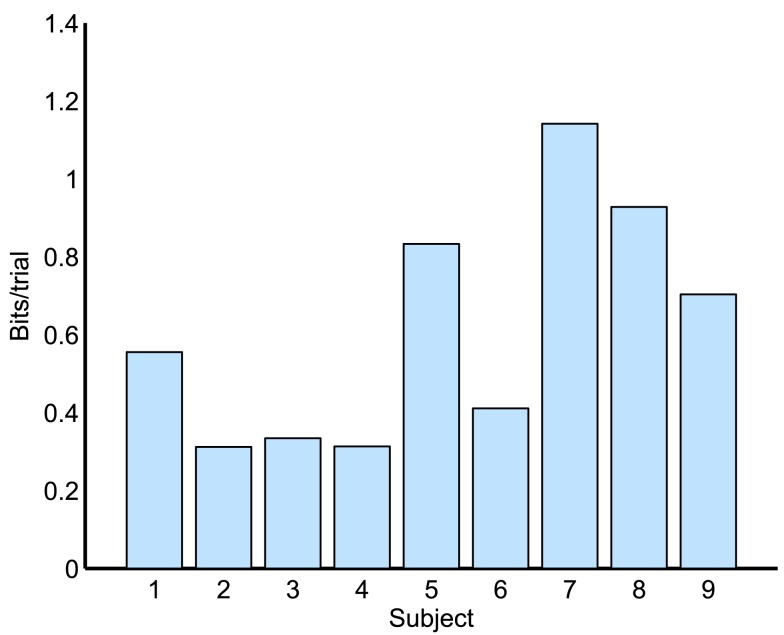
**Information transfer rate (ITR) obtained for all nine subjects**.

Bit rate is an objective measure for measuring improvement in a BCI and for comparing different BCIs (Wolpaw et al., 2002). Bit rate for four different choices is shown as bits/trial in Figure 7 for each subject. By comparing Table 4 and Figure 7, it can be seen that the bit rate is in direct proportion to kappa score.

### Performance estimation for stage removal and artifact removal

**Figure 8:**
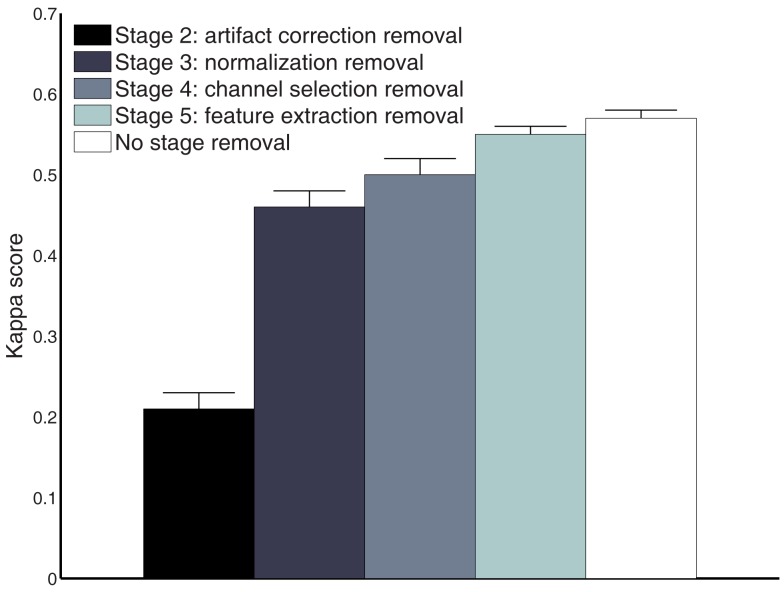
**Comparison of the performance measures (kappa scores) for different processing stage removal across all subjects on the test set (session 2)**.

**Figure 9:**
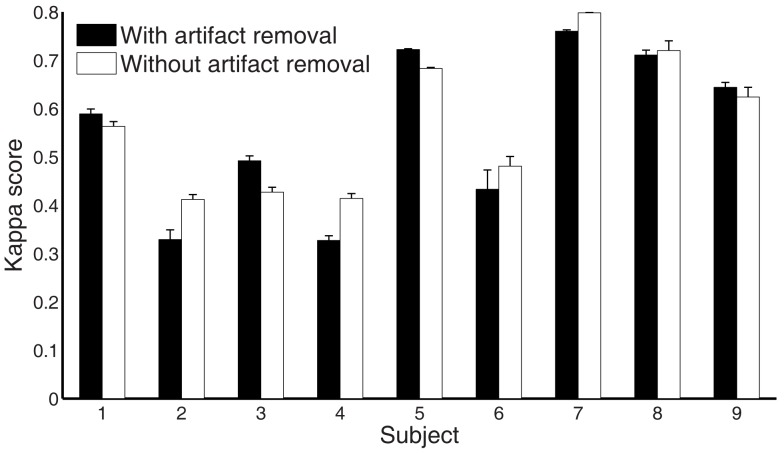
**Comparison of the performance measures (kappa scores) for the whole processing framework with and without artifact removal for subject 1 to 9 on the test set (session 2)**.

To further evaluate each stage’s performance of the processing framework separately, we reported kappa scores for each subject on the test set by removing the different stages rather than adding them (as done in Table 3). Figure 8 shows that stages 2–5 contribute to the final performance of the whole framework, especially stage 2. Furthermore, we present the classification performances comparison of the whole processing framework with and without artifact removal in Figure 9. We observed that there is only a slight improvement in classification performance in subject1, 3, 5, and 9 when removing artifact-contaminated trials from training and test set. There was no significant performance difference between methods with and without artifact removal.

## Conclusion

Implementing information exchange between humans and machines through the use of EEG signals is one of the biggest challenges in signal processing and biomedical engineering and one of the fundamental issues is the proper interpretation of EEG signals (Kolodziej et al., 2010).

This paper illustrates how the proposed processing framework decodes the EEG signal for multi-class mental tasks. Our robust brain-computer interface processing framework FSDE includes five stages. Basically, we focused on two challenges for online EEG decoding. One was the EOG artifact correction and other artifacts separation. Considering raw EEG signals contaminated by not only EOG artifacts but also artifacts generated by other sources, we extended the work of Schlögl et al. (2007), proposed our new computational model, and implemented the automatic artifact correction method for recovering the original source signals. The other was the channel selection based on the reactive non-contiguous discriminating frequency sub-bands instead of setting a broad frequency range which was proposed by Pfurtscheller and Lopes da Silva (1999). We did not find any similar studies in the literatures. In Arvaneh et al. (2011), the EEG data were band-pass filtered using a manually selecting frequency range, i.e., 8–35 Hz. In Ang et al. (2008), the multi-channel EEG signals were first band-pass-filtered into multiple frequency bands (4–8 Hz, 8–12 Hz, …, 36–40 Hz), then the authors extracted common spatial patterns (CSP) features from each of these bands. Compared to these existing methods, our method introduced an automatic selection of subject-specific reactive frequency sub-bands through the training session and we confirmed that non-contiguous band filtering approach provides a much more accurate way to quantify the sensorimotor rhythms in the frequency domain. During training, the method was computationally intensive, but there was almost no computational time cost in test session. In BCI applications, ITR is used for evaluating the system performance. And we did obtain good performances that were comparable to that of the winner of the competitions but using different methods. We would also like to point out that our method has another clear advantage over previous methods. Indeed, from a Neuroscience point of view, it is often of interest to find which brain signals can explain behavior. Our method automatically provides the set of signals (electrodes and frequency bands) that lead to the best prediction of behavior. This is extremely valuable for experimental research involving EEG.

The proposed method was evaluated using a publicly available dataset of BCI competition. Using the same criterion (i.e., kappa score), the overall four-class kappa values were comparable to other models but without requiring any artifact-contaminated trial removal. The performance also showed that multi-class MI tasks can be reliably discriminated using a few selected channels. This may be a promising avenue for fast online and robust EEG-based BCI applications.

## Conflict of Interest Statement

The authors declare that the research was conducted in the absence of any commercial or financial relationships that could be construed as a potential conflict of interest.

## Footnotes

^1^BCI Competition IV http://www.bbci.de/competition/iv/

^2^http://www.cis.hut.fi/projects/ica/fastica/

^3^http://biosig.sourceforge.net/

^4^http://www.csie.ntu.edu.tw/~cjlin/libsvm

^5^The results were announced on November 2008. The top rankers at http://www.bbci.de/competition/iv/results/index.html#dataset2a

## Appendix

### FastICA

Independent component analysis is a method for determining underlying factors or components from multivariate statistical data. What distinguishes ICA from other methods is that it looks for components that are both statistically independent, and non-Gaussian (Hyvarinen et al., 2001). The FastICA algorithm (Hyvarinen and Oja, 1997; Hyvärinen, 1999) is a commonly used ICA algorithm which uses a fixed-point iteration scheme to extract independent components by separately maximizing the negentropy of each mixture. It is a computationally more efficient method for performing the estimation of ICA than conventional gradient descent methods for ICA. To estimate *W*, these steps were followed:

1. Calculate the mean *M* of the data *Y* for each row.
2. Y←Y-M.
3. Calculate PCA of *Y*, returns the eigenvector (*E*) and diagonal eigenvalue (*D*) matrices.
4. Calculate the whitening matrices $\text{WM}\;:\;\text{WM}\leftarrow D^{-1∕2}E^{T}.$
5. Whiten Y:Y←WM⋅Y.
6. Begin calculating the ICA using Hyvarinen’s fixed-point algorithm (Hyvarinen and Oja, 1997; Hyvärinen, 1999) for each channel:
  - Initialize the weight matrix ω and set ε;
  - Repeat
    (a) Update the function *g*, which is the derivative of the function *G* used in the general contrast function: $\omega_{\text{new}}^{+}\leftarrow E\left\{yg\left({\omega_{\text{old}}}^{T}y\right)\right\}-E\left\{g^{\prime }\left({\omega_{\text{old}}}^{T}y\right)\right\}\omega_{\text{old}}.$
    (b) Normalize: $\omega_{\text{new}}\leftarrow \omega_{\text{new}}^{+}∕∥\omega_{\text{new}}^{+}∥.$
    (c) Until $∥\omega_{\text{new}}-\omega_{\text{old}}∥<\varepsilon .$
7. Return *W* = [ω_1_, ω_2_, …, ω_*n*_]^T^, where *n* denotes the number of channels.

### Sample entropy

For a given one-dimensional time series of *T* points: *S *= {s(1), s(2), …, s(T)}, computation of SampEn is shown in the following steps:

1. Change *S* into T–*m *+ 1 vectors:
  $S_{m}\left(i\right)=\left[s\left(i\right),s\left(i+1\right),\cdots \;,s\left(i+m-1\right)\right]$ for *i* = 1, …, *T *− *m* + 1, where *m* denotes the length of sequences to be compared.
2. Define the distance between each of two vectors as: $d\left[S_{m}\left(i\right),S_{m}\left(j\right)\right]=\underset{0\leq k\leq m-1}{\max}|s\left(i+k\right)-s\left(j+k\right)|$ for *i,j = *1, …, *N* − *m*, and *i *= *j*.
3. Given *r* (the tolerance for accepting matches), $B_{i}^{m}\left(r\right)$ is defined as 1/(*T – m*) times the number of vectors S_m_(*j*) falling within vector distance *r* of *S_m_*(*i*), 1 ≤ *j ≤* *T – m; j *≠ *i*,
  $$B^{m}\left(r\right)=\frac{1}{T-m}\overset{N-m}{\underset{i=1}{\sum }}B_{i}^{m}\left(r\right).$$
4. Similarly, calculate $B_{i}^{m+1}\left(r\right)$ which is defined as 1/(*T –* *m – *1)times the number of vectors *S*_m+1_(*j*) falling within vector distance *r* of *S*_m+1_(*i*), 1(*i*), 1 ≤ *j ≤* *T – m – 1*,
  $$B^{m+1}\left(r\right)=\frac{1}{T-m-1}\overset{N-m-1}{\underset{i=1}{\sum }}B_{i}^{m+1}\left(r\right).$$
5. Calculate the Sample entropy (SampEn) as
  $$\text{SampEn}\left(m,r\right)=\underset{N\to \infty }{\lim}\left[-\ln\left(\frac{B^{m+1}\left(r\right)}{B^{m}\left(r\right)}\right)\right].$$
